# 基于定量指纹图谱技术的经典名方清骨散基准样品量值传递分析

**DOI:** 10.3724/SP.J.1123.2022.09024

**Published:** 2023-02-08

**Authors:** Xin XU, Tong WEI, Qianqian XUE, Jiahao AI, Guixin LI, Zhongguo LIU, Dan LI, Jincai HOU, Hongli JIN, Yanfang LIU, Xinmiao LIANG

**Affiliations:** 1.赣江中药创新中心，江西 南昌 330000; 1. Ganjiang Chinese Medicine Innovation Center，Nanchang 330000，China; 2.江西省中药药效物质基础重点实验室，江西 南昌 330000; 2. Jiangxi Provincial Key Laboratory for Pharmacodynamic Material Basis of Traditional Chinese Medicine，Nanchang 330000，China; 3.中国科学院大连化学物理研究所，辽宁 大连 116023; 3. Dalian Institute of Chemical Physics，Chinese Academy of Sciences，Dalian 116023，China; 4.神威药业集团有限公司，河北 石家庄 051430; 4. Shineway Pharmaceutical Group Co.，Ltd.，Shijiazhuang 051430，China

**Keywords:** 定量指纹图谱, 量值传递, 清骨散, 基准样品, quantitative fingerprint, quality value transfer, Qinggusan, reference sample

## Abstract

清骨散为第一批《古代经典名方目录》中的第69方，现代临床常用于治疗非感染性发热。但目前关于清骨散基准样品及其量值传递的研究较少，制约了其复方制剂的研发与上市，因此亟须建立一种全面、准确的质量控制方法，阐明关键质量属性。通过制备15批清骨散基准样品，明确干膏率范围，建立基准样品高效液相色谱（HPLC）定量指纹图谱测定方法并进行相似度评价，采用高效液相色谱-四极杆-飞行时间质谱（HPLC-Q-TOF-MS）技术对特征峰进行指认与归属，结合指标成分龙胆苦苷、芒果苷、胡黄连苷Ⅱ、胡黄连苷Ⅰ和甘草酸的含量范围及转移率范围进行量值传递分析。结果表明，15批清骨散基准样品的干膏率为24.10%~26.88%，指纹图谱相似度均大于0.95，对12个共有峰进行指认，包括6个环烯醚萜类、2个黄酮类、1个生物碱类、1个三萜皂苷类以及2个其他类，其中5个成分来源于胡黄连，3个成分来源于秦艽，2个成分来源于知母，1个成分来源于银柴胡，1个成分来源于甘草；15批基准样品中指标成分的含量范围分别为龙胆苦苷17.92~27.55 mg/g、芒果苷1.83~4.42 mg/g、胡黄连苷Ⅱ 23.08~36.44 mg/g、胡黄连苷Ⅰ 8.43~15.04 mg/g、甘草酸0.94~2.39 mg/g，从饮片到基准样品的转移率分别为龙胆苦苷47.91%~63.95%、芒果苷22.96%~59.39%、胡黄连苷Ⅱ 60.82%~77.82%、胡黄连苷Ⅰ 64.25%~99.53%、甘草酸15.30%~39.30%。该研究通过清骨散基准样品定量指纹图谱与质谱定性技术相结合，基本明确其化学组成，进行量值传递研究，阐明关键质量属性，为清骨散复方制剂的质量控制提供依据。

## Abstract (en)

Qinggusan is the 69th prescription in the first batch of “Catalogue of Ancient Chinese Classic Formulas”. In modern clinical practice， Qinggusan is mainly used to treat noninfectious fever. However， because few studies on Qinggusan reference samples and their quality value transfer are available， the development and promotion of its compound preparations are restricted. Therefore， establishing an accurate and comprehensive quality control method to clarify the critical quality attributes of Qinggusan reference samples is of great importance. In this study， 15 batches of Qinggusan reference samples were processed to determine the range of their dry extract ratios. Quantitative high-performance liquid chromatography （HPLC） fingerprint analysis was performed using a Waters Symmetry Shield RP18 column （250 mm×4.6 mm， 5 μm） with acetonitrile-0.1% （v/v） formic acid aqueous solution as the mobile phase in gradient elution mode. The flow rate was 1.0 mL/min， the column temperature was 30 ℃， and the detection wavelength was 254 nm. The HPLC fingerprints of the Qinggusan reference samples were established under these conditions to evaluate their similarity. The established method was systematically validated and found to demonstrate good precision， repeatability， and sample stability. Subsequently， characteristic peaks were identified and attributed by HPLC-quadrupole-time-of-flight-mass spectrometry （HPLC-Q-TOF-MS） analysis. MS was performed in electrospray ionization mode， the data were collected in both positive- and negative-ion modes， and the detection range was *m/z* 50-2000. The contents and transfer rate ranges of the index components， namely， gentiopicrin， mangiferin， picroside Ⅱ， picroside Ⅰ， and glycyrrhizic acid， were determined to analyze the quality value transfer of the samples. The results demonstrated that the dry extract rate of the 15 batches of Qinggusan reference samples ranged from 24.10% to 26.88% and that their fingerprint similarities were generally greater than 0.95. Twelve common peaks were identified by reference identification， literature comparison， and high-resolution MS analysis. Twelve compounds， including six iridoid glycosides， two flavonoids， one alkaloid， one triterpenoid saponin， and two others. Among them， L-picein， androsin， picroside Ⅳ， picroside Ⅱ and picroside Ⅰ were from Picrorhizae Rhizoma， loganin acid， swertiamarin and gentiopicrin were from Gentianae Macrophyllae Radix， neomangiferin and mangiferin were from Anemarrhenae Rhizoma， dichotomine B was from Stellariae Radix， and glycyrrhizic acid was from Glycyrrhizae Radix et Rhizoma. The five key components presented good linear relationships in their respective linear ranges， and all correlation coefficients were higher than 0.999. The relative standard deviations （RSDs） of precision， stability， and repeatability were less than 1.3%. The average recoveries varied between 95.92% and 102.5%， with RSDs less than 3.9%； these values meet the requirements defined in the 2020 edition of the Chinese Pharmacopoeia. The contents of gentiopicrin， mangiferin， picroside Ⅱ， picroside Ⅰ， and glycyrrhizic acid in the 15 batches of reference samples were in the range of 17.92-27.55， 1.83-4.42， 23.08-36.44， 8.43-15.04， and 0.94-2.39 mg/g， respectively， and their transfer rates from the decoction pieces to the reference samples were 47.91%-63.95%， 22.96%-59.39%， 60.82%-77.82%， 64.25%-99.53%， and 15.30%-39.30%， respectively. In this study， the chemical components of Qinggusan reference samples were comprehensively identified and their quality value transfer was studied through the combination of HPLC fingerprinting and MS. Clarification of the critical quality attributes of Qinggusan reference samples could provide a basis for the quality control of Qinggusan compound preparations.

清骨散（QGS）出自明代王肯堂所著的《证治准绳》，由银柴胡（YCH）、胡黄连（HHL）、秦艽（QJ）、醋鳖甲（CBJ）、地骨皮（DGP）、青蒿（QH）、知母（ZM）、甘草（GC）8味药组成，具有清虚热、退骨蒸之功效，用于治疗肝肾阴虚、虚火内扰证，证见骨蒸潮热或低热日久不退等^[1]^。现代临床常用于外科术后持续性发热、肺结核发热、晚期癌性发热等非感染性发热的治疗^[2⇓-4]^。

2018年4月，国家中医药管理局会同国家药品监督管理局公布了第一批《古代经典名方目录》^[5]^，清骨散为第69号方。近期国家药监局药审中心发布的《按古代经典名方目录管理的中药复方制剂药学研究技术指导原则（试行）》（以下简称“指导原则”）^[6]^中明确提出，基准样品作为复方制剂药学研究的参照，是经典名方开发的重要一环，其中量值传递研究是基准样品研究的关键；应建立经典名方基准样品整体质量评价方法，加强化学成分表征，开展量值传递研究以明确关键质量属性。然而，目前关于清骨散基准样品及其量值传递研究鲜有报道，制约了经典名方清骨散复方制剂的研发。

2008年，本课题组首次提出基于定量指纹图谱技术的中药质量控制策略^[7,8]^。定量指纹图谱技术是中药指纹图谱技术与多指标成分定量分析相结合的中药质量控制模式，突出指标性成分的同时，兼顾了微量成分及中药整体质量，使得中药质量控制更加快速、全面、准确可靠，目前已经在20多个中药材和30多个中成药质量控制方面得到了广泛的应用^[9⇓-11]^。本实验根据清骨散关键信息考证结果，采用随机数表法组合制备出15批清骨散基准样品冻干粉，建立了清骨散基准样品高效液相色谱-二极管阵列检测（HPLC-DAD）定量指纹图谱，并利用高效液相色谱-四极杆-飞行时间质谱（HPLC-Q-TOF-MS）技术对指纹谱图中特征峰进行指认，进一步采用定量指纹图谱方法测定了其中5个主要活性成分的含量，分析饮片-基准样品的量值传递规律，明确关键质量属性，为经典名方清骨散的复方制剂研究及质量标准制定奠定基础。

## 1 实验部分

### 1.1 仪器、试剂与材料

Waters Alliance e2695高效液相色谱系统，包括四元泵、DAD检测器、自动进样器、柱温箱、Empower色谱工作站（Waters，美国）； Agilent Infinity Ⅱ 1290-6545高效液相色谱-四极杆飞行时间质谱仪（Agilent，美国）； XS105型十万分之一分析天平（梅特勒-托利多仪器上海有限公司）。

对照品龙胆苦苷（批号：110770-201918，规格：20 mg，纯度：97.10%）、芒果苷（批号：111607-201704，规格：20 mg，纯度：98.10%）、胡黄连苷Ⅱ（批号：111596-201805，规格：20 mg，纯度：93.20%）、胡黄连苷Ⅰ（批号：111727-201702，规格：20 mg，纯度：95.60%）、甘草酸铵（批号：110731-202021，规格：20 mg，纯度：96.20%）购自中国食品药品检定研究院。色谱级乙腈、甲醇、乙醇均购自美国Sigma-Aldrich公司，色谱级甲酸和磷酸购自上海阿拉丁生化科技股份有限公司。实验室用水来自Milli-Q超纯水净化系统（Millipore，美国）。

清骨散处方药材由神威药业有限公司提供，经中国科学院大连化学物理研究所杨小平高级工程师鉴定，8味药材均符合2020年版《中国药典》的相关规定。根据各药味炮制历史沿革考证信息，制成饮片，将各批饮片随机组合，按照《古代经典名方中药复方制剂及其物质基准的申报资料要求（征求意见稿）》^[12]^要求，制备15批清骨散基准样品。具体组方药材信息见表1。

**表1:** 清骨散组方药材信息

Medicinal material	Base resource	Medicinal part	Geographical origins
Stellariae Radix （YCH）	*Stellaria dichotoma* L. var. *lanceolata* Bge.	dried radix root	5 batches from Tongxin，Ningxia；5 batches from Pingluo，Ningxia；5 batches from Guyuan，Ningxia
Anemarrhenae Rhizoma（ZM）	*Anemarrhena asphodeloides* Bge.	dried rhizome	5 batches from Anguo，Hebei；5 batches from Bozhou，Anhui；5 batches from Zhangjiakou，Hebei
Picrorhizae Rhizoma （HHL）	*Picrorhiza scrophulariiflora* Pennell	dried rhizome	5 batches from Dangxiong，Xizang；5 batches from Shigatse，Xizang；5 batches from Shannancuona，Xizang
Lycii Cortex（DGP）	*Lycium chinense* Mill.	dried radix root cortex	5 batches from Yuncheng，Shanxi；5 batches from Julu，Hebei；5 batches from Longxi，Gansu
Gentianae Macrophyllae Radix（QJ）	*Gentiana macrophylla* Pall.	dried radix root	5 batches from Zhashui，Shanxi；5 batches from Longxian，Shanxi；5 batches from Zhengning，Gansu
Artemisiae annuae Herba（QH）	*Artemisia annua* L.	dried aerial part	5 batches from Yongzhou，Hunan；5 batches from Qichun，Hubei；5 batches from Xiangfan，Hubei
Trionycis carapax（CBJ）	*Trionyx sinensis* Wiegmann	dried carapace	5 batches from Wuhan，Hubei；5 batches from Xiaoxian，Anhui；5 batches from Yueyang，Hunan
Glycyrrhizae Radix et Rhizoma（GC）	*Glycyrrhiza uralensis* Fisch.	dried root and rhizome	5 batches from Minqin，Gansu；5 batches from Longxi，Gansu；5 batches from Tacheng，Xinjiang

### 1.2 基准样品的制备

根据国家中医药管理局发布的《古代经典名方目录（第一批）》^[5]^中清骨散处方和制法，经关键信息考证，明代一钱折合成现代3.73 g，一盅折合成现代200 mL，确定处方为银柴胡5.60 g，知母、胡黄连、地骨皮、秦艽、青蒿、醋鳖甲各3.73 g，甘草1.87 g；制备方法为取8味处方量饮片共29.85 g，置陶瓷锅中，加水400 mL，浸泡30 min，煎煮至160 mL，滤过，减压浓缩至投料量和浓缩液质量比为1∶1.6，冷冻干燥，即得清骨散基准样品冻干粉。

### 1.3 干膏率的测定

取1.2节中制备的清骨散水煎液，精密量取水煎液25 mL，置于已干燥至恒重的蒸发皿中，水浴蒸干后，于105 ℃干燥3 h，置于干燥器中冷却30 min，迅速精密称定重量，计算干膏率。干膏率=*WV/vY*×100%，其中*W*表示所得干膏质量（g）， *V*表示水煎液总体积（mL）， *v*表示取样体积（mL）， *Y*表示处方饮片质量（g）。

### 1.4 对照品溶液的制备

取龙胆苦苷、芒果苷、胡黄连苷Ⅱ、胡黄连苷Ⅰ和甘草酸铵对照品适量，精密称定，加50%乙醇水溶液制成每1 mL含龙胆苦苷200 μg、芒果苷20 μg、胡黄连苷Ⅱ 200 μg、胡黄连苷Ⅰ 50 μg、甘草酸铵20 μg的混合溶液。

### 1.5 供试品溶液的制备

取清骨散基准样品冻干粉约0.15 g，精密称定，置具塞锥形瓶中，精密加入50%乙醇水溶液25 mL，密塞，称定重量，超声处理（功率400 W，频率40 kHz）15 min，放冷，再称定重量，用50%乙醇水溶液补足减失的重量，摇匀，滤过，取续滤液。

### 1.6 分析条件

#### 1.6.1 HPLC-DAD

色谱柱：Waters Symmetry Shield RP18（250 mm×4.6 mm， 5 μm）；流动相：A相为乙腈，B相为0.1%甲酸水溶液。梯度洗脱条件：0~8 min， 0A； 8~10 min， 0A~10%A； 10~32 min， 10%A~12%A； 32~43 min， 12%A~15%A； 43~50 min， 15%A~16%A； 50~78 min， 16%A~20%A； 78~109 min， 20%A~52%A。流速：1 mL/min；进样量：10 μL；柱温：30 ℃；检测波长：254 nm。

#### 1.6.2 HPLC-Q-TOF-MS

HPLC-Q-TOF-MS的色谱条件同1.6.1节。

离子源：电喷雾双喷离子源（Dual AJS ESI），正、负离子采集模式；扫描模式：一级质谱采集（MS），自动二级质谱采集（auto MS/MS）；扫描范围：MS *m/z* 50~2000， MS/MS *m/z* 50~2000；气帘气温度：320 ℃；鞘气温度：320 ℃；干燥气流速：8 L/min，离子化压力：+3500 V/-3500 V；毛细管出口电压：75 V；碰撞能：40 eV。

## 2 结果与讨论

### 2.1 干膏率

按照1.3节中的方法测定干膏率，结果显示15批清骨散基准样品的干膏率在24.10%~26.88%之间，均值为25.10%，干膏率在均值的95.78%~107.10%，符合“指导原则”关于干膏率应控制在均值90%~110%的要求。

### 2.2 指纹图谱研究

#### 2.2.1 指纹图谱的建立

取15批（S1~S15）清骨散基准样品冻干粉，按1.5节中的方法制备基准样品供试品溶液，按1.6.1节中的HPLC-DAD色谱条件依次进样检测，获得15批清骨散基准样品的HPLC指纹图谱，将S1~S15批清骨散基准样品的色谱图全部导入《中药色谱特征图谱相似度评价系统软件》（2012版），以S1为参照谱图，采用中位数法进行自动匹配，加以多点校正，得到15批清骨散基准样品的指纹图谱叠加图（图1），确定12个共有色谱峰，生成对照指纹图谱（图2）。

**图1:**
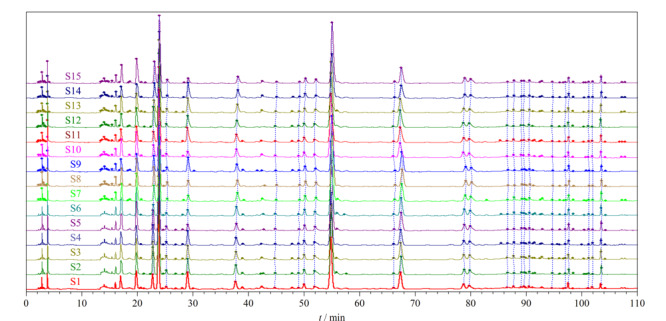
15批清骨散基准样品的HPLC指纹图谱

**图2:**
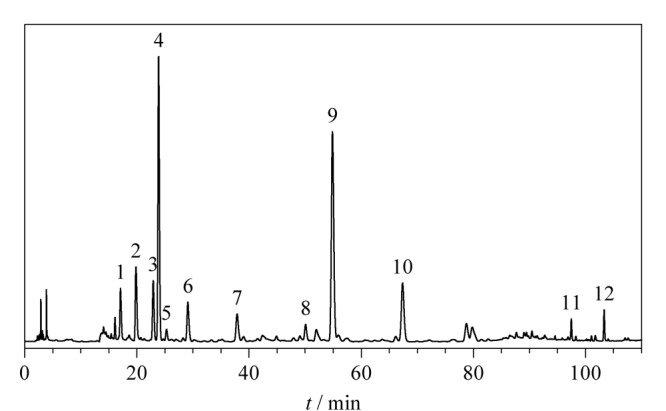
清骨散基准样品的HPLC对照指纹图谱

采用1.6.2节中的HPLC-Q-TOF-MS的条件对清骨散基准样品供试品溶液及各味饮片水煎液的化学组成进行定性分析。通过与对照品对比、一级精确质量数及二级碎片离子信息分析，对清骨散基准样品对照指纹图谱中12个共有峰进行指认^[13⇓⇓⇓⇓-18]^，包括6个环烯醚萜类、2个黄酮类、1个生物碱类、1个三萜皂苷类以及2个其他类（表2）。

**表2:** 清骨散基准样品特征峰的鉴定

No.	*t*_R_/ min	Formula	Adduct ion	Theoretical mass （*m/z*）	Experimental mass（*m/z*）	Mass error/ ppm	MS^2^（*m/z*）	Identification	Medicine
1	17.043	C_16_H_24_O_10_	[M-H]^-^	375.1297	375.1298	0.27	151.0761，113.0241，59.0140	loganin acid^[13]^	QJ
		C_14_H_18_O_7_	[M+HCOO]^-^	343.1035	343.1039	1.17	135.0451，120.0227	L-picein^[14]^	HHL
2	20.530	C_16_H_22_O_10_	[M+HCOO]^-^	419.1195	419.1197	0.48	141.0194，101.0243，89.0243， 71.0139，59.0141	swertiamarin^[13]^	QJ
		C_15_H_20_O_8_	[M+HCOO]^-^	373.1140	373.1170	8.04	165.0558，150.0323	androsin^[14]^	HHL
3	22.866	C_25_H_28_O_16_	[M+H]^+^	585.1450	585.1452	0.34	369.0599，327.0499，303.0498， 273.0387	neomangiferin^[15]^	ZM
4^*^	23.885	C_16_H_20_O_9_	[M+HCOO]^-^	401.1189	401.1176	-3.24	149.0607，113.0243，71.0139， 59.0140	gentiopicrin^[13]^	QJ
5	25.267	C_14_H_12_N_2_O_4_	[M+H]^+^	273.0870	273.0870	0	227.0813，209.0707，181.0758， 169.0757，154.0649	dichotomine B^[16,17]^	YCH
6	29.135	/	[M-H]^-^	/	353.0890	/	/	/	HHL
7^*^	37.815	C_19_H_18_O_11_	[M+H]^+^	423.0922	423.0922	0	351.0496，339.0498，327.0501， 273.0392，303.0499，285.0393， 273.0392	mangiferin^[15]^	ZM
8	50.980	C_24_H_28_O_12_	[M-H]^-^	507.1558	507.1514	-8.68	163.0398，145.0292，119.0498， 117.0342	picroside Ⅳ^[14]^	HHL
9^*^	54.774	C_23_H_28_O_13_	[M-H]^-^	511.1457	511.1453	-0.82	207.0660，167.0348，152.0111， 123.0450	picroside Ⅱ^[14]^	HHL
10^*^	67.260	C_24_H_28_O_11_	[M+HCOO]^-^	537.1614	537.1641	5.03	147.0453，103.0551，97.0293	picroside Ⅰ^[14]^	HHL
11	97.516	/	[M+HCOO]^-^	/	753.4022	/	707.4059，689.3950	/	HHL
12^*^	103.348	C_42_H_62_O_16_	[M-H]^-^	821.3965	821.3952	-1.58	351.0565，193.0350，175.0252	glycyrrhizic acid^[18]^	GC

ppm：10^-6^；* compared with standards.

环烯醚萜类化合物 环烯醚萜类化合物主要包括环戊烷型、环戊烯型、环氧烷型、裂环型等4类成分。环烯醚萜类化合物在负离子模式下主要以[M-H]^-^、[M+HCOO]^-^的准分子离子峰形式存在，其中环氧烷型环烯醚萜类主要发生糖苷键以及酯键的断裂^[13,14]^，以胡黄连苷Ⅱ为例，保留时间为54.774 min，根据负离子模式下，一级质谱信息显示，其准分子离子峰为*m/z* 511.1453 [M-H]^-^，进一步进行二级质谱扫描，由准分子离子峰中酯键断裂得到碎片离子*m/z* 167.0348 [M-H-Glc-C_9_H_10_O_4_]^-^，再进一步丢失1分子CO_2_得到碎片离子*m/z* 123.0450 [M-H-Glc-C_9_H_10_O_4_-CO_2_]^-^，并与对照品比对，质谱数据一致，故确定其为胡黄连苷Ⅱ，根据裂解规律，以此类推，清骨散基准样品中共鉴定出6个环烯醚萜类化合物，包括表2中化合物马钱苷酸（loganin acid）、獐牙菜苦苷（swertiamarin）、龙胆苦苷（gentiopicrin）、胡黄连苷IV（picroside Ⅳ）、胡黄连苷Ⅱ（picroside Ⅱ）、胡黄连苷Ⅰ（picroside Ⅰ）。

黄酮类化合物 黄酮类化合物在正离子模式下主要以[M+H]^+^的准分子离子峰形式存在，主要发生糖链、侧链的裂解和脱水^[15]^，以芒果苷为例，保留时间为37.815 min，根据正离子模式下，一级质谱信息显示，其准分子离子峰为*m/z* 423.0922 [M+H]^+^，进一步进行二级质谱扫描，由准分子离子峰中葡萄糖裂解得到碎片离子*m/z* 303.0499 [M+H-C_4_H_8_O_4_]^+^，再进一步丢失一分子CH_2_O生成*m/z* 273.0392 [M+H-C_4_H_8_O_4_-CH_2_O]^+^。另一个裂解特征是准分子离子峰*m/z* 423.0922 [M+H]^+^直接脱去2分子H_2_O和2分子CH_2_O得到碎片离子*m/z* 327.0501 [M+H-C_2_H_6_O_4_]^+^。与对照品比对，质谱数据一致，故确定其为芒果苷。根据裂解规律，以此类推，清骨散基准样品中共鉴定出2个黄酮类化合物，包括表2中化合物新芒果苷（neomangiferin）和芒果苷（mangiferin）。

生物碱类化合物 吲哚类生物碱在正离子模式下主要以[M+H]^+^的准分子离子峰形式存在，主要发生环内叔胺碳氮键断裂^[16,17]^，以银柴胡胺B为例，保留时间为25.267 min，根据正离子模式下，一级质谱信息显示，其准分子离子峰为*m/z* 273.0870 [M+H]^+^，进一步进行二级质谱扫描，由准分子离子峰中季胺发生*α*键断裂及丢失1分子CO_2_得到碎片离子*m/z* 169.0757 [M+H-C_3_H_4_O_4_]^+^，再进一步发生环内叔胺碳氮键断裂丢失NH生成*m/z* 154.0649 [M+H-C_3_H_4_O_4_-NH]^+^。另一个裂解特征是准分子离子峰*m/z* 273.0870 [M+H]^+^丢失一分子CH_2_O_2_得到碎片离子*m/z* 227.0813 [M+H-CH_2_O_2_]^+^，再丢失一分子H_2_O得到碎片*m/z* 209.0707 [M+H-CH_2_O_2_-H_2_O]^+^，烯醇结构不稳定，进一步丢失一分子CO得到碎片离子*m/z* 181.0758 [M+H-CH_2_O_2_-H_2_O-CO]^+^，鉴定该化合物为银柴胡胺B（dichotomine B）。

#### 2.2.2 指纹图谱方法学考察

精密度试验 取同一批次清骨散基准样品，制备供试品溶液，连续进样6次，记录指纹图谱。各特征峰的相对保留时间RSD均小于1%，主要特征峰的相对峰面积RSD小于5%，表明仪器精密度良好。

稳定性试验 取同一批次清骨散基准样品，制备供试品溶液，分别于0、6、12、18、24 h注入液相色谱仪，记录指纹图谱。各特征峰的相对保留时间RSD均小于1%，主要特征峰的相对峰面积RSD小于5%，表明供试品溶液24 h内稳定性良好。

重复性试验 取同一批次清骨散基准样品，制备6份供试品溶液，进样，记录指纹图谱。各特征峰的相对保留时间RSD均小于1%，主要特征峰的相对峰面积RSD小于5%，表明该方法重复性良好。

#### 2.2.3 指纹图谱相似度评价

将得到的15批清骨散基准样品指纹图谱导入《中药色谱特征图谱相似度评价系统软件》（2012版），选择响应较高且出峰时间相对居中的胡黄连苷Ⅱ作为参照峰，进行色谱峰匹配，计算不同批次清骨散基准样品与对照指纹图谱的相似度。结果显示，15批清骨散基准样品的相似度均>0.95，表明清骨散基准样品的制备工艺稳定，批间质量一致性较好。

#### 2.2.4 指纹图谱共有峰归属

通过清骨散基准样品S1与银柴胡、地骨皮、胡黄连、青蒿、知母、醋鳖甲、秦艽、甘草8味饮片的指纹图谱比对（图3），将清骨散基准样品中12个共有峰归属到各味饮片。其中1、2号峰来源于秦艽和胡黄连，3、7号峰来源于知母，4号峰来源于秦艽，5号峰来源于银柴胡，6、8~11号峰来源于胡黄连，12号峰来源于甘草。进一步计算各味饮片与清骨散基准样品的指纹图谱相似度，依次为0.79（胡黄连）、0.61（秦艽）、0.11（知母）、0.07（甘草）、0.02（银柴胡）、0（地骨皮）、0（青蒿）、0（鳖甲）， 3味饮片对基准样品指纹图谱均无贡献度。研究表明，地骨皮和青蒿水提液中含有微量的地骨皮乙素等生物碱类、异绿原酸A/B/C等苯丙素类成分，针对该类化合物带电荷的特点，建议可以探索基于离子交换模式选择性富集目标化合物，开发鉴别方法。鳖甲的主成分为脯氨酸等氨基酸类、糖类，该类化合物极性强，在反相色谱上保留较弱，建议可以探索大类成分质控方法。因此，后续工作将通过清骨散基准样品指纹图谱特征峰归属，结合鉴别、大类成分测定等方法，实现全方药味的整体质量控制，阐明各药味特征成分的量值传递规律。

**图3:**
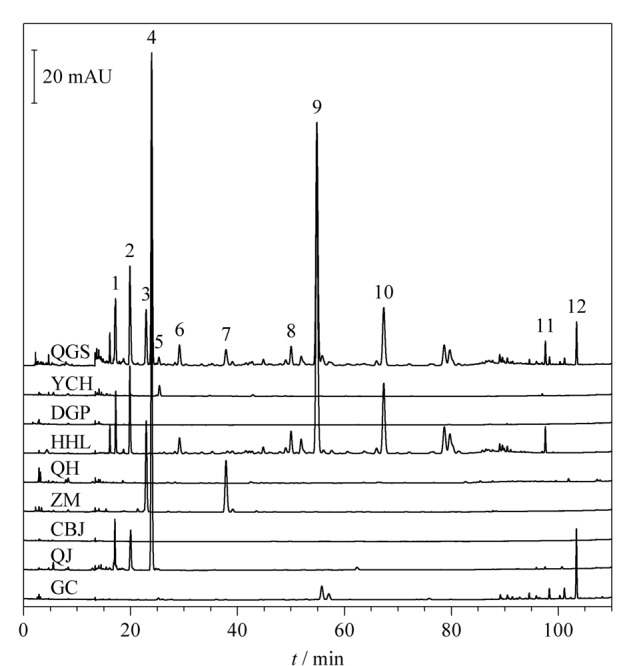
清骨散基准样品S1与单味饮片的HPLC指纹图谱

### 2.3 指标成分含量测定及量值传递研究

根据文献研究，清骨散的现代药理作用主要为解热、抗炎和调节免疫功能^[19,20]^，其主要活性成分为君药银柴胡中银柴胡胺B^[16]^，臣药知母中芒果苷^[21]^，臣药胡黄连和佐药秦艽中环烯醚萜类^[22,23]^、包括胡黄连苷Ⅱ、胡黄连苷Ⅰ、龙胆苦苷等，使药甘草中甘草酸^[24,25]^。采用指纹图谱结合高分辨质谱技术，以上类别中6个活性成分得到指认，并且可以从饮片传递到基准样品。其中，银柴胡胺B的含量低于万分之一，不宜作为定量指标。因此，最终选择芒果苷、胡黄连苷Ⅱ、胡黄连苷Ⅰ、龙胆苦苷和甘草酸为定量指标成分，建立清骨散基准样品的含量测定方法。

#### 2.3.1 系统适用性试验

取龙胆苦苷、芒果苷、胡黄连苷Ⅱ、胡黄连苷Ⅰ和甘草酸的混合对照品溶液，按照1.6.1节中的HPLC-DAD色谱条件进行测定，记录色谱图（见图4）。龙胆苦苷、芒果苷、胡黄连苷Ⅱ、胡黄连苷Ⅰ和甘草酸5种化学成分的保留时间分别为24.39、39.11、55.82、68.35、103.47 min，本实验条件下所测的5种成分均实现基线分离，拖尾因子均在0.95~1.05，理论塔板数大于10000，系统适用性试验良好。

**图4:**
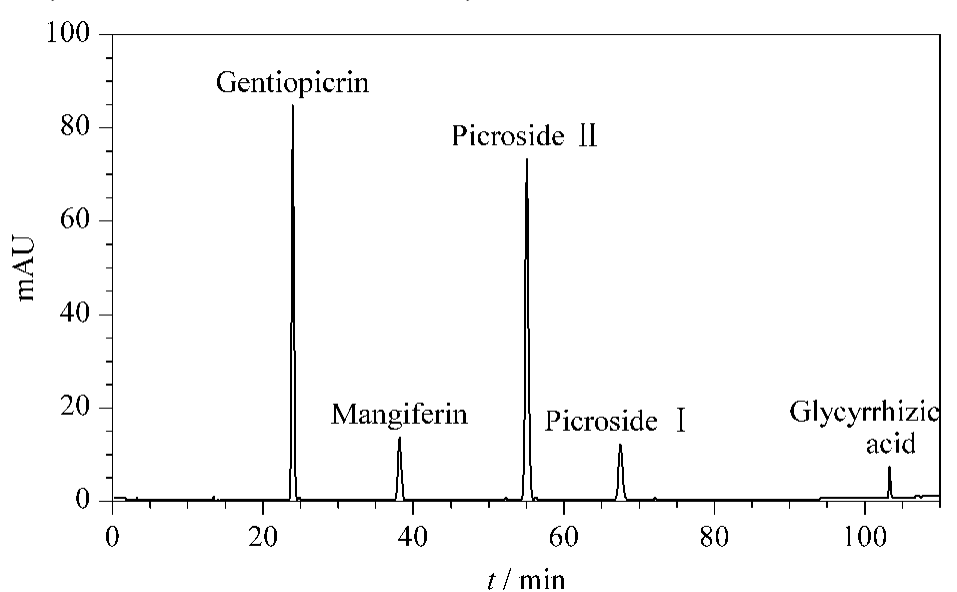
混合对照品的HPLC-DAD色谱图

#### 2.3.2 方法学考察

线性关系 精密吸取龙胆苦苷、芒果苷、胡黄连苷Ⅱ、胡黄连苷Ⅰ和甘草酸对照品储备液适量，配制系列混合对照品溶液，按照1.6.1节中的HPLC-DAD色谱条件进行测定，以各对照品质量浓度*X*（mg/L）为横坐标，峰面积*Y*为纵坐标，绘制标准曲线，得线性回归方程、相关系数（*R*^2^）和线性范围，结果见表3。

**表3:** 5个成分的线性方程、相关系数、线性范围、检出限及定量限

Compound	Linear equation	*R*^2^	Linear range/（mg/L）	LOD/（mg/L）	LOQ/（mg/L）
Gentiopicrin	*Y*=1.062×10^4^*X*+2.934×10^4^	0.9994	24.72-247.2	0.1360	0.4533
Mangiferin	*Y*=4.095×10^4^*X*-8.598×10^2^	0.9993	3.997-39.97	0.0581	0.1938
Picroside Ⅱ	*Y*=9.961×10^3^*X*+3.833×10^4^	0.9993	35.13-351.3	0.2237	0.7457
Picroside Ⅰ	*Y*=2.336×10^4^*X*+1.533×10^4^	0.9994	9.972-99.72	0.3307	1.1023
Glycyrrhizic acid	*Y*=8.126×10^3^*X*+3.679×10^3^	0.9994	3.750-37.50	0.1099	0.3662

*Y*：peak area；*X*：mass concentration，mg/L.

检出限和定量限 精密吸取龙胆苦苷、芒果苷、胡黄连苷Ⅱ、胡黄连苷Ⅰ和甘草酸对照品储备液适量，逐级稀释后进行测定，按照1.6.1节中的HPLC-DAD色谱条件进行测定，以3倍信噪比（*S/N*=3）计算检出限（LOD）， 10倍信噪比计算定量限（LOQ），结果表明该方法具有良好的灵敏度，结果见表3。

精密度试验 取同一批次清骨散基准样品，制备供试品溶液，连续进样6次，计算龙胆苦苷、芒果苷、胡黄连苷Ⅱ、胡黄连苷Ⅰ和甘草酸含量的RSD，均在0.20%~0.31%之间，表明仪器精密度良好。

稳定性试验 取同一批次清骨散基准样品，制备供试品溶液，分别于0、6、12、18、24 h注入液相色谱仪，计算龙胆苦苷、芒果苷、胡黄连苷Ⅱ、胡黄连苷Ⅰ和甘草酸含量的RSD，均在0.27%~0.40%之间，表明供试品溶液24 h内稳定性良好。

重复性试验 取同一批次清骨散基准样品，制备6份供试品溶液，进样，计算龙胆苦苷、芒果苷、胡黄连苷Ⅱ、胡黄连苷Ⅰ和甘草酸含量的RSD，均在0.37%~1.3%之间，表明该方法重复性良好。

加标回收试验 精密称取9份已知含量的同一批次清骨散基准样品，每份0.075 g，均分为3组，每组分别按5个成分含量的50%、100%、150%的比例精密加入相应的龙胆苦苷、芒果苷、胡黄连苷Ⅱ、胡黄连苷Ⅰ和甘草酸对照品，按1.5节中的方法制备供试品溶液，按1.6.1节中的HPLC-DAD色谱条件进行测定，结果显示，龙胆苦苷、芒果苷、胡黄连苷Ⅱ、胡黄连苷Ⅰ和甘草酸的平均回收率均在95%~105%之间，RSD均在1.5%~3.9%之间，表明方法准确度良好。

#### 2.3.3 样品含量测定及量值传递研究

依据2020年版《中国药典》对饮片中5个指标成分进行含量测定，采用已开发的定量指纹图谱方法对15批清骨散基准样品进行分析，测定龙胆苦苷、芒果苷、胡黄连苷Ⅱ、胡黄连苷Ⅰ和甘草酸含量（见表4），并计算饮片→基准样品中指标成分转移率。转移率=*wm/WM*×100%，其中*w*表示基准样品中指标成分的含量，*m*表示基准样品的样品量，*W*表示饮片中指标成分的含量，*M*表示处方中饮片质量。结果显示，15批清骨散基准样品中龙胆苦苷、胡黄连苷Ⅱ、胡黄连苷Ⅰ的含量及转移率均在其均值的70%~130%范围内，表明清骨散基准样品制备工艺稳定。然而，部分基准样品中甘草酸和芒果苷的含量和转移率未在其均值的70%~130%之间。研究表明，甘草和知母不同组织部位中指标成分含量存在显著性差异^[26,27]^，是造成不同批次基准样品中甘草酸和芒果苷含量差异较大的主要原因。因此，建议制定甘草和知母饮片内控质量标准，从源头保证饮片质量均一，以确保清骨散基准样品的质量稳定。

**表4:** 清骨散基准样品中指标成分含量及转移率

No.	Gentiopicrin				Mangiferin				Picroside Ⅱ				Picroside Ⅰ				Glycyrrhizic acid		
	*w*_RS_/ （mg/g）	*w*_DP_/ （mg/g）	*r*_t_/ %		*w*_RS_/ （mg/g）	*w*_DP_/ （mg/g）	*r*_t_/ %		*w*_RS_/ （mg/g）	*w*_DP_/ （mg/g）	*r*_t_/ %		*w*_RS_/ （mg/g）	*w*_DP_/ （mg/g）	*r*_t_/ %		*w*_RS_/ （mg/g）	*w*_DP_/ （mg/g）	*r*_t_/ %
S1	23.82	85.74	55.00		2.79	18.64	31.60		31.12	105.23	60.82		11.39	36.48	64.25		1.78	24.85	28.68
S2	21.20	80.53	56.77		3.10	17.11	39.07		30.50	107.78	61.21		13.10	39.63	71.41		1.23	25.63	20.37
S3	25.10	87.11	59.91		2.60	15.95	35.27		25.60	76.54	68.90		11.60	29.74	80.62		1.15	20.86	22.23
S4	20.33	82.70	57.91		2.30	17.63	30.53		34.54	107.88	73.60		11.50	40.07	65.97		1.75	28.29	28.97
S5	18.27	75.30	50.14		2.46	18.71	28.13		34.19	98.65	71.51		13.35	28.00	98.35		1.78	25.69	28.81
S6	23.61	93.85	52.91		1.87	17.66	22.96		25.52	77.13	68.75		10.56	27.64	79.49		1.40	22.60	25.63
S7	17.92	84.30	47.91		1.83	15.86	25.15		33.65	107.30	62.76		14.93	38.34	82.23		1.79	25.52	30.58
S8	18.88	72.87	61.33		3.87	19.69	43.25		33.06	101.64	70.78		11.55	37.44	67.10		2.39	26.36	38.91
S9	21.23	79.31	58.38		2.83	17.91	36.05		25.09	75.72	72.34		11.64	29.08	87.41		1.34	22.66	25.37
S10	27.55	94.69	63.95		4.42	16.47	59.39		33.89	95.87	77.82		13.27	28.81	99.53		1.81	28.38	28.88
S11	22.93	80.80	50.23		3.19	14.64	45.27		26.10	73.24	71.99		10.14	26.54	77.27		1.90	23.70	33.58
S12	21.77	93.00	61.97		2.25	19.09	27.32		23.08	75.64	67.69		8.43	25.44	73.39		2.17	24.60	39.30
S13	18.94	85.13	48.11		2.72	16.32	34.62		34.52	97.34	71.82		10.11	30.05	68.17		1.61	23.20	27.93
S14	26.24	95.84	57.37		1.99	16.89	25.70		36.44	101.64	75.21		15.04	29.89	99.28		0.94	24.98	15.30
S15	24.97	103.61	52.32		3.07	17.66	38.37		34.62	101.94	71.97		13.12	30.00	92.49		2.25	27.76	36.25
Average value	22.19	86.32	55.61		2.75	17.35	34.84		30.97	93.56	69.81		11.98	31.81	80.46		1.69	25.01	28.72
Fluctuation	17.92-	72.87-	86.15-		1.83-	14.64-	65.89-		23.08-	73.24-	87.12-		8.43-	25.44-	79.85-		0.94-	20.86-	53.27-
range	27.55	103.61	114.98		4.42	19.69	170.44		36.44	107.88	111.48		15.04	40.07	123.69		2.39	28.38	136.83

*w*_RS_：content in reference sample；*w*_DP_：content in decoction piece；*r*_t_：transfer rate.

## 3 结论

本研究建立了一种经典名方清骨散基准样品的HPLC-DAD定量指纹图谱方法，结合HPLC-Q-TOF-MS技术，指认了12个共有峰，归属到5味饮片，并通过5个主要活性成分的含量测定阐释了饮片→基准样品的量值传递规律。该方法具有简便、准确可靠等特点，可对经典名方清骨散基准样品进行定性和定量分析，明确清骨散基准样品关键质量属性，为后续清骨散复方制剂的开发及质量控制提供依据。
